# Late-life affective disorders and risk of progression to dementia: A retrospective cohort study of patients in secondary care

**DOI:** 10.1192/bjp.2025.45

**Published:** 2025-03-31

**Authors:** Laith Alexander, Katherine Beck, Mariia Bocharova, Allan H. Young, Robert Stewart, Rowena Carter, Christoph Mueller

**Affiliations:** 1 Institute of Psychiatry, Psychology and Neuroscience, King’s College London, London, UK; 2 South London and Maudsley NHS Foundation Trust, London, UK

## Abstract

**Background:**

Late-life affective disorders (LLADs) are common and are projected to increase by 2050. There have been several studies linking late-life depression to an increased risk of dementia, but it is unclear if bipolar affective disorder or anxiety disorders pose a similar risk.

**Aims:**

We aimed to compare the risk of LLADs progressing to all-cause dementia, and the demographic and clinical variables mediating the risk.

**Methods:**

We used the South London and the Maudsley NHS Foundation Trust (SLaM) Clinical Records Interactive Search (CRIS) system to identify patients aged 60 years or older with a diagnosis of any affective disorder. Cox proportional hazard models were used to determine differences in dementia risk between late-life anxiety disorders *vs*. late-life depression, and late-life bipolar disorder *vs*. late-life depression. Demographic and clinical characteristics associated with the risk of dementia were investigated.

**Result:**

5,695 patients were identified and included in the final analysis. 388 had a diagnosis of bipolar affective disorder, 1,365 had a diagnosis of an anxiety disorder and 3,942 had a diagnosis of a depressive disorder. Bipolar affective disorder was associated with a lower hazard of developing dementia compared to depression (adjusted model including demographics and baseline cognition, HR: 0.60; 95% CI: 0.41-0.87). Anxiety disorders had a similar hazard of developing dementia (adjusted HR: 1.05; 95% CI: 0.90-1.22). A prior history of a depressive disorder reduced the risk of late-life depression progressing to a dementia – suggesting the new onset of a depressive disorder in later life is associated with higher risk – but a prior history of anxiety disorders or bipolar affective disorder did not alter risk.

**Conclusions:**

LLADs have a differential risk of developing all-cause dementia, with clinically relevant factors influencing the risk. Further prospective cohort studies are needed to explore the link between LLADs and dementia development, and mediators of the lower risk of dementia associated with late-life bipolar disorder compared to late-life depression.

## Introduction

Dementia is a significant complication of late-life affective disorders LLADs, and its association has been most extensively studied in late-life depression (1). Depression is also the most common mental disorder in late life, affecting 2% of those over the age of 60 (2) and is projected to increase significantly by 2050 (3). Depression, especially in late-life, has been associated with up to a two-fold increased risk for dementia (4–6). The development of a LLAD if less than 3 months follow-up was available, or the repeated relapse of a pre-existing illness, might accelerate biological ageing and promote earlier neurodegeneration (7,8), therefore increasing the risk of dementia. Alternatively, affective symptoms may herald the development of a dementia, as a psychological symptom that develops during the progressive and protracted period of cognitive decline.

The link between dementia and LLADs other than depression, including anxiety and bipolar affective disorder, is less well understood. Regarding anxiety disorders, a prior meta-analysis has suggested that anxiety disorders increase the risk of dementia and a stronger association is evident for anxiety symptoms developing in later life (9). A second meta-analysis replicated the finding of an increased risk of dementia associated with anxiety disorders in patients aged ∼60-80 years old (10). However, there is heterogeneity across individual studies, with some not demonstrating a link (11) or highlighting that the increased risk is mediated by concurrent depressive symptoms (12).

A link between bipolar affective disorder and dementia dates as far back as 1913, when Kraepelin wrote on the autopsy of a patient with manic depression, noting widespread arteriosclerosis of blood vessels (13). However, there is marked variability in recent studies. A recent meta-analysis suggested psychotic disorders (including bipolar affective disorder) were associated with subsequent dementia, although evidence was “sparse” (1). Some studies have found evidence of cognitive impairments which are worse with later onset of bipolar affective disorder (14), whereas others have suggested age and illness duration are negatively correlated with the degree of cognitive impairment (15). Other studies still have emphasised that there is marked intra-individual short-term variability in cognitive impairments (16), whereas some have identified similar age-related patterns of cognitive decline to healthy controls (17,18) suggesting that the disorder “does not have a significant adverse impact on cognitive and brain aging” (17).

Previous work has tried to identify biomarkers and risk factors for LLADs (particularly depression) progressing to dementia. Poor antidepressant response (19), disease phenotype, higher symptom burden (20), physical health measures such as hand-grip strength (21) and genetic markers (22,23) have all provided some insight but not enough to be clinically actionable. Furthermore, it remains unclear as to whether the biomarkers and risk factors differ across different LLADs.

No study to date has directly compared the risk of dementia progression across LLADs, nor have they determined the demographic and clinical features which might mediate differences in risk. This study aims to address this gap by leveraging electronic health records using a retrospective cohort design. Using late-life depression as a reference group with a well-established risk of dementia progression, we explore whether anxiety disorders and bipolar affective disorder similarly increase dementia risk. Additionally, we aim to identify demographic and illness-related characteristics that may modulate the risk of dementia progression in these disorders. By addressing these questions, this research seeks to refine our understanding of the relationship between LLADs and dementia, inform clinical risk stratification, and guide interventions to mitigate the cognitive and functional decline associated with LLADs.

## Methods

### Data source

The study cohort was generated using the Clinical Record Interactive Search (CRIS) resource (24). CRIS was developed in 2008 to provide access to de-identified structured and open-text data extracted from the electronic health records system of the South London and Maudsley NHS Foundation Trust (SLaM). SLaM is one of Europe’s largest mental health and dementia care providers, serving the 1.4 million residents of four south London boroughs (Croydon, Lambeth, Lewisham and Southwark). CRIS records reflect the total secondary care mental health usage of this population, with the anonymised records of over 500,000 mental health and dementia patients. Records from primary care are not included.

CRIS has research ethical approval as an anonymised database for secondary analysis (Oxford Research Ethics Committee C, reference 23/SC/0257). Data are extracted from structured fields or free text (events, clinical correspondence), for which a wide range of natural language processing (NLP; ‘text-mining’) applications are used (25). All NLP algorithms and their performance data are listed on an open-source online catalogue (26).

### Sample

To be included patients needed to have a diagnosis of one of the following affective disorders according to ICD-10 (27) criteria: bipolar affective disorder (F30 or F31), depressive disorder (F32 or F33), or anxiety disorder (F41). The diagnosis needed to be made between 1st January 2008 and 31st December 2022 and patients needed to be at least 60 years old at the time of diagnosis. The date of first recording of a LLAD served as index date for longitudinal analyses. If a patient had several LLAD diagnoses recorded at the time of diagnoses we prioritised bipolar affective disorder over anxiety disorder, and anxiety disorder over depressive disorder. Patients were excluded if a diagnosis of dementia was made before the LLAD diagnosis or within three months of the diagnosis, as this is the usual time frame in which memory services establish dementia diagnoses. Patients were considered to have a previous diagnosis of bipolar affective disorder, depression or anxiety disorder if such a diagnosis could be identified before the index date. Further, a patient was considered to have a previous diagnosis of depression if their diagnosis at index date was recurrent depressive disorder (ICD-10: F33).

### Outcome

We ascertained whether a diagnosis of dementia according to ICD-10 criteria (F00-F03) was made in the follow-up period. Patients were followed up from the first affective disorder diagnosis bold = p<0.05 (index date) to either the first recording of a dementia diagnosis, their last attended appointment with a SLaM clinician, or a census date on 17th March 2023. Patients were excluded less than 3 months of follow-up was available.

### Covariates and potential predictors of dementia development

Data were extracted on age at affective disorder diagnosis, gender, ethnicity (classified as White, Black, Asian, other), and neighbourhood-level deprivation score (Index of Multiple Deprivation) (28). We also extracted the score of Health of the Nation Outcome Scales (HoNOS) (29) which are routinely used in UK mental health services. We used the following subscales as recorded closest to first affective disorder diagnosis: agitated behaviour, non-accidental self-injury (which includes suicidal ideation), drug/alcohol problems, hallucinations or delusions, depressed mood, physical illness or disability, cognitive problems, and difficulties with activities of daily living (ADLs), and problems with social relationships. HoNOS items are rated from 0 (least severe status) to 4 (most severe status). For this analysis, we dichotomised the subscales and considered scores of 0-1 as the patient not experiencing problems in the domain, while scores of 2-4 indicated that problems were present. To measure physical health more accurately, the HoNOS physical illness or disability scale was divided in three categories: ‘no or minor’ (Score 0-1), ‘mild’ (Score 2), and ‘moderate to severe’ (Score 3-4). HoNOS scales have been shown to be related to dementia risk in other CRIS cohorts (30).

### Statistical analysis

Data were analysed using STATA 15 software (31). First, we generated descriptive statistics comparing those with bipolar affective disorder in later life to those with depression in later life and comparing those with anxiety disorder in later life to those with depression. Then we used adjusted and unadjusted Cox proportional hazard models to determine differences in dementia risk between bipolar affective disorder or anxiety disorder and depression. As a sensitivity analysis, we further compared bipolar affective disorder or anxiety disorder to those with late-onset depressive disorder (i.e. those without a prior history of depression). We then used the adjusted Cox proportional hazard model to determine predictors of dementia development in each of the three affective disorder groups. The adjusted models were adjusted for age at affective disorder diagnosis, age squared, gender, ethnicity, marital status, deprivation, baseline cognition and time*cognition interaction. Adjusting for baseline cognition accounted for pre-existing cognitive differences that may confound the relationship between LLADs and dementia risk.

Using the test of Schoefield residuals we tested whether the proportional hazards assumption was met. This assumption was violated for the HoNOS cognitive problems and the HoNOS physical disabilities scales. Therefore, a time*cognition interaction and/or a time*physical illness interaction was included in the relevant analyses.

In the whole dataset 25% of patient had data on at least one covariate missing. Variables with missing data were deprivation, ethnicity, HoNOS scores, and marital status. Therefore, we used the mi package in STATA to create 25 imputed datasets, which were generated by replacing missing values with simulated values assembled from covariates and outcome values without missing data, as well as additional prescribing information available (32). Rubin’s rules (33) were applied to combine coefficients in final analyses.

## Results

We identified 11,192 patients with an affective disorder diagnosis after the age of 60 years. After removing 5,947 patients as they developed dementia within three months (or before affective disorder diagnosis) or had less than 3 months of follow-up time available, the final sample consisted of 5,695 patients. Of these 388 (6.8%) had a diagnosis of bipolar affective disorder, 1,365 (24.0%) an anxiety disorder, and 3,942 (69.2%) a depressive disorder. In total 29.1% of patients with bipolar affective disorder had a prior history of the condition, 5.3% of patients with anxiety disorder had a prior history of an anxiety disorder, and 30.3% of patients with depression had a prior history of depression.

### Baseline characteristics (Table 1)

Characteristics at the time of first affective disorder diagnosis recording after the age of 60 years are presented in Table 1.

### Bipolar affective disorder vs. Depression

Compared to those with depression, patients diagnosed with bipolar affective disorder were younger at the point of affective disorder diagnosis, from less deprived areas, and a higher proportion presented with agitated behaviour or hallucinations and/or delusions. There were no significant differences in cognitive difficulties at bipolar/depression diagnosis. Patients with bipolar affective disorder less frequently presented with non-accidental self-injury (which includes suicidal ideation), depressed mood, moderate-to-severe physical health problems, and problems with activities of daily living.

### Anxiety vs. Depression

When compared to patients with depression, patients with anxiety disorders were at a similar age at the time of the affective disorder diagnosis, but were more frequently female, of White ethnicity, married, and from less deprived areas. A lower proportion of patients with anxiety disorders were from a Black background, and fewer patients presented with agitated behaviour, drug/alcohol problems, hallucinations and/or delusions, depressed mood, or cognitive problems. Patients with anxiety disorders less frequently had moderate-severe physical health problems, and problems with activities of daily living or social relationships.

### Risk of dementia

Of 388 patients with bipolar affective disorder, 29 (7.5%) developed dementia; of 1,365 patients with anxiety disorder, 232 (17.0%) developed dementia; and of 3,942 patients with depression, 701 (17.8%) developed dementia. Mean follow-up time (until dementia diagnosis/death/census) was 4.0 (SD +/- 3.3) years for bipolar affective disorder, 2.8 (+/- 2.7) years for anxiety disorders, and 2.9 (+/- 3.0) years for depression. The available follow-up time for bipolar affective disorder was significantly longer than for anxiety or depression (one way ANOVA, p<0.001; bipolar affective disorder *vs.* anxiety and bipolar affective disorder *vs.* depression, p<0.001; anxiety *vs* depression, p=0.317). The ANOVA refers to the f/u time not the proportion developing dementia.

Compared to depression, patients with bipolar affective disorder had a lower risk of developing dementia in unadjusted analysis (HR: 0.30; 95% confidence interval (CI): 0.21-0.44) and in the adjusted analysis (HR: 0.60; 95% CI: 0.41-0.87).

Compared to depression, patients with anxiety had a similar dementia risk in unadjusted analysis (HR: 1.01; 95% CI: 0.87-1.17) and in the adjusted analysis (HR: 1.05; 95% CI: 0.90-1.22).

In a sensitivity analysis, we excluded recurrent depressive disorder to ascertain whether the onset of a new depressive disorder in later-life confers particular risk. In unadjusted analysis, both bipolar affective disorder (HR 0.25; 95% CI 0.17-0.37) and anxiety disorders (HR 0.81; 95% CI 0.69-0.94) had a comparatively lower risk of dementia, suggesting the development of new depression in late life might confer greater risk. However, whilst the effect survived adjustment for bipolar affective disorder (HR 0.52; 95% CI 0.35-0.76), it did not for anxiety disorders (HR 0.94; 95% CI 0.81-1.10).

### Factors associated with dementia development in the three sub-cohorts (Table 2)

Using multivariate Cox proportional hazards model, we determined the factors associated with subsequent dementia development in each group. The results are shown in Table 2.

In patients with bipolar affective disorder, hallucinations and/or delusions and cognitive problems measured close to the time of affective disorder diagnosis were associated with a higher risk of dementia development. Neither age at diagnosis nor a prior history of bipolar affective disorder were associated with dementia development.

In patients with anxiety disorder, age at anxiety disorder diagnosis and cognitive problems were associated with a higher risk of dementia development, while depressed mood, hallucinations and delusions, and moderate-severe physical health problems yielded a lower risk. A previous history of an anxiety disorder did not affect dementia risk.

In patients with depression, age at depression diagnosis, Black and ‘Other’ (compared to White) ethnicity, and cognitive problems yielded a higher risk of developing dementia. The presence of non-accidental self-injury, difficulties with social relationships, and a previous history of depression was associated with a lower risk of developing dementia.

## Discussion

In a large cohort of patients diagnosed with an affective disorder in later life in routine mental health services, we evaluated how the risk of progressing to a dementia diagnosis differed between bipolar affective disorder, anxiety disorders, and depression. While patients with late-life anxiety disorders had a similar dementia risk as patients with late-life depression, patients with late-life bipolar affective disorder had a 40% lower dementia risk than those with depression after confounder adjustment. These findings advance our understanding of how distinct affective disorder subtypes influence dementia risk, and by identifying specific variables associated with increased risk, our work could help refine risk prediction models for dementia among patients with affective disorders enabling early identification of high-risk individuals. It provides a basis for targeted interventions that address modifiable factors, potentially mitigating the progression to dementia.

### Dementia development

The reduced rate of dementia diagnosis in those with late-life bipolar affective disorder compared to those with late-life depression (HR: 0.60; 95% CI: 0.41-0.87) occurred despite the longer follow-up time available for these patients. One explanation might relate to the substantially younger age at the time of affective disorder diagnosis for bipolar affective disorder patients, compared to those with depression. However, age and age-squared were adjusted for in our multivariate model, and the risk remained lower. Furthermore, age did not seem to predict dementia development in people diagnosed with bipolar affective disorder. A second possibility is that patients with bipolar affective disorder who survive to dementia-risk ages are relatively healthier than those with depression, owing to the survival pressure associated with a severe mental illness (‘survivor effect’). Therefore, the cohort reaching this age range may carry a relatively lower baseline dementia risk. A third reason may be that bipolar affective disorder is more distinguishable from dementia compared to depression, which can present with marked cognitive impairment (34).

The distinguishability of bipolar affective disorder relates to a fourth reason – access to treatment. Many older people with depression do not access treatment, and the risk of progression to dementia is higher in untreated depression (35). As bipolar affective disorder is arguably more distinguishable and apparent, patients may be more likely to access appropriate treatment. Cognitive decline in bipolar affective disorder seems to stabilise over time, especially in people who are receiving treatment (36); by contrast, cognitive decline continues to progress in patients with unipolar depression (37). Lithium may be important – previous work has suggested that bipolar affective disorder is associated with an increased risk of dementia, but that the risk is reduced when patients are treated with lithium (38). Due to low numbers, we were unable to determine if there were different patterns for patients on or off lithium. Retrospective cohort studies suggest that lithium exposure (across both unipolar and bipolar depression) reduces the risk of developing dementia subsequently (39). A randomised clinical trial investigating the utility of lithium in protecting against the progressive cognitive decline associated with mild cognitive impairment has shown that lithium improves long-term global cognitive outcomes (40). The presence of some patients on lithium in the cohort we examined might therefore result in an overall lower risk of dementia in patients with late-life bipolar affective disorder compared to those with late-life depression, although this cannot be disentangled from the potential survivor effect, or baseline health differences. Furthermore, some patients with late-life depression may be on lithium augmentation as treatment.

The presence of a late-life anxiety disorder was associated with a similar risk of dementia to the presence of a late-life depressive disorder (HR: 1.02; 95% CI: 0.88-1.19). In some studies, anxiety disorders have been shown to increase the risk of all-cause dementia (10,41); however, other studies have not supported a link (11). The emotional dysregulation characteristic of anxiety disorders may be due to neurobiological changes in vulnerable brain regions associated with dementia, such as the hippocampus, which precede the onset of cognitive decline (21). Anxiety disorders are associated with a higher risk of cardiovascular disease (42), raised levels of glucocorticoids (43), and avoidant behaviour and social isolation, all of which may also act to increase the risk of dementia. It is also important to consider diagnostic clarity; there is overlap between anxiety and depression and the disorders are highly co-morbid, especially in later life (44,45). This might contribute to the similar dementia risk.

### Factors associated with the development of dementia

In those with a depressive disorder, a prior history seemed to reduce the risk of a subsequent diagnosis of dementia. This might seem counterintuitive; previous studies have suggested that no matter when a depressive episode occurs, it increases the risk of dementia (46), and that repeated depressive episodes increase the risk of subsequently developing dementia (47,48) through the cumulative effects of sustained stress and a failure of improvement in cognition between episodes. One interpretation might be that the *new* onset of a depressive disorder later in life is more likely to reflect a dementia prodrome rather than a primary affective illness, compared to the relapse of a pre-existing depressive disorder (20). Therefore, a prior history of the disorder would be protective. This is corroborated by our finding that in an unadjusted sensitivity analysis, excluding recurrent depression, *both* bipolar affective disorder and anxiety disorders were associated with a lower risk of dementia compared to late-life depression. This further adds evidence that the new onset of a depressive disorder in late-life is particularly associated with the development of subsequent dementia (21).

Interestingly, problems with social relationships in those with late-life depression were associated with a lower risk of dementia. The HoNOS subscale however refers to relationship problems – which are risk factors for depression (as lack of intimacy or poor social support) – rather than social isolation in the context of cognitive decline (29,49). Problematic relationships on this scale may therefore be better predictors of a depressive illness rather than an underlying dementia.

In late-life depression, Black (and Other) ethnicity was associated with an increased risk of dementia progression. Dementia incidence is higher in people of Black ethnicity, likely contributed to by socioeconomic and physical health factors (in particular, vascular risk factors; (50)). The increased burden of psychiatric symptoms, greater degree of functional impairment and poorer access to timely mental health treatment associated with late-life depressive (51) and anxiety (52) disorders in people of Black ethnicity may contribute to the increased risk of subsequent progression to dementia, through the effects of chronic stress and/or incomplete cognitive recovery between episodes. There may also be an increased burden of physical illness and comorbidity in people of Black ethnicity, which additionally increase the risk of dementia (53).

In those with anxiety disorders, the presence of co-morbid depressed mood was protective against dementia. Similarly, the presence of hallucinations and/or delusions was associated with a lower risk. In older people, isolated anxiety, especially if severe, can suggest an organic process such as dementia. By contrast, anxiety in the context of other symptoms may be occurring in conjunction with another psychiatric disorder; anxiety in older people is often noted in the context of a depressive illness (44) and generalised anxiety in particular is frequently considered to be a manifestation of the same underlying depressive illness. Moderate-severe physical health issues yielded a lower dementia risk too; this may be a mortality effect.

In those with late-life bipolar affective disorder, hallucinations, delusions and cognitive problems were predictors of the subsequent development of dementia. The presence of hallucinations and delusions indicates a worse disease severity, resulting in hospitalisation and poor concordance with medications for both mental and physical illness (54), all of which could contribute to an increased dementia risk. It is also possible that psychotic symptoms might herald the development of dementia, not occurring infrequently in, for example, Alzheimer’s disease (55) and Lewy body dementias (56). Hallucinations and delusions have been linked to a ‘mild behavioural impairment’ which has been described as a transitional state towards dementia, analogous to mild cognitive impairment (57). It is also possible that a misdiagnosed delirium accounts for the increased risk associated with these symptoms (58).

### Strengths and Limitations

Our findings are derived from data collected using CRIS, which leverages the largest and most detailed electronic health record system for secondary mental health care globally. This enhances the generalisability of our results, particularly to urban populations engaged in secondary care mental health services, given the ethnic and socioeconomic diversity of the catchment area. The depth of CRIS data allows for diagnostic ascertainment and exploration of confounding factors, bolstering the credibility of our findings.

However, limitations must be acknowledged. As our cohort is drawn exclusively from secondary care, it may not fully capture individuals with milder affective disorders or those managed entirely in primary care or community settings. Additionally, while the diversity of the population is a strength, findings may not directly generalise to rural populations, or to healthcare systems outside of the NHS.

Selection bias arises as our patients are selected from a predominantly secondary care cohort. Many late-life depressive and anxiety disorders may be managed at a primary care level. Cases of depression and anxiety in secondary care are typically more severe than those seen in primary care; bipolar affective disorder is almost always managed in secondary care. In our cohort, the risk of dementia in secondary care depression and anxiety may therefore be higher than that seen in primary care settings.

Information bias may arise from inaccuracies in the clinical records. Misclassification errors related to diagnosis are possible, although we believe this is a relatively small threat; in CRIS, diagnoses are made by specialised psychiatrists who are applying ICD-10 criteria (compared to primary care or national registries). The misclassification of bipolar affective disorder is lower in later life, given the more extensive past psychiatric history and reduced challenge of disentangling bipolar affective disorder and a personality disorder. The risk of misclassification with anxiety disorders *vs*. unipolar depression may be higher, given that anxiety in later-life often occurs in the context of a depressive illness (44,45). The comparison between anxiety disorders *vs*. unipolar depression may, therefore, in part be a comparison between depression with anxiety *vs*. depression without anxiety.

Patients with co-morbid late-life depression and late-life anxiety were classified in the anxiety disorders group; it was not possible to disentangle the primary diagnosis in these cases and this may affect the comparative rates of dementia in the groups. There may be ascertainment bias as we may have missed some patients’ previous histories of bipolar affective disorder and are therefore underestimating their occurrence; indeed, the sample size of patients with late-life bipolar affective disorder was small. Finally, the HoNOS illness variables might be inaccurately recorded.

Inherent in the retrospective cohort design is the issue of confounders. We tried to control for demographic confounders in our multivariate modelling. However, factors such as different ages of the cohorts, lifestyle factors and physical health comorbidities may not be completely controlled for using multivariate modelling. The HoNOS subscales do not include diagnostic information about specific physical health conditions, such as diabetes and cardiovascular disease; including such information could more comprehensively account for physical health as a potential confounder.

Causality cannot be established in a study of this design. It is also challenging to establish a clear temporal relationship between the exposures (late-life affective disorders) and the outcome (dementia), as cognitive impairments and neuropathological changes may have predated the onset of late-life affective disorders but may not have been detected (59).

## Conclusion

In this large cohort of over 5,000 patients with an LLAD, late-life anxiety disorders were associated with a similar risk of progression to dementia as late-life depressive disorders, whereas late-life bipolar affective disorder was associated with a lower risk. We were able to determine predisposing and protective factors, within LLAD cohorts, associated with the risk of dementia. Prospective cohort studies are needed to further explore the link between LLADs and the subsequent risk of developing dementia, including whether these populations are particularly at risk of certain dementia subtypes.

## Figures and Tables

**Table 1:** Baseline characteristics

Baseline characteristics	Bipolar affective disorder (n=388)	Anxiety disorder (n=1,365)	Depression (n=3,942)	P-value (Bipolar vs. depression)	P-value (Anxiety vs. depression)
*Sociodemographic characteristics*					
Mean age at affective disorder diagnosis (years, SD)	66.5 (6.8)	72.3 (8.7)	72.2 (9.1)	<**0.001**	0.779
Female gender (%) Ethnicity (%)	53.9%	67.0%	57.4%	0.176	<**0.001**
White	75.8%	82.9%	76.4%	0.776	<**0.001**
Black	14.2%	7.4%	12.5%	0.353	<**0.001**
Asian	5.7%	6.0%	7.2%	0.291	0.146
Other	4.3%	3.7%	3.9%	0.666	0.673
Married or cohabiting (%)^a^	31.0%	36.1%	32.3%	0.620	**0.019**
Mean index of deprivation (SD)^a^	27.0 (10.9)	27.1 (10.8)	28.2 (10.8)	**0.047**	**0.001**
*HoNOS mental and physical health problems* ^a,b^					
Agitated behaviour	34.0%	14.1%	13.9%	<**0.001**	0.887
Non-accidental self-injury	4.8%	8.0%	14.8%	<**0.001**	<**0.001**
Drug/alcohol problems	11.1%	5.3%	8.5%	0.099	<**0.001**
Hallucinations and/or delusions	23.3%	7.1%	12.9%	<**0.001**	<**0.001**
Depressed mood	33.3%	59.5%	74.4%	<**0.001**	<**0.001**
Cognitive problems	19.9%	18.9%	21.7%	0.431	**0.036**
*HoNOS Physical illness or disability* (%)^a^					
No or minor	53.9%	45.6%	38.4%	<**0.001**	<**0.001**
Mild	27.7%	28.0%	27.6%	0.957	0.784
Moderate - severe	18.4%	26.4%	34.0%	<**0.001**	<**0.001**
*HoNOS functional problems* (%)^a,b^					
Activities of daily living	30.5%	35.2%	43.0%	<**0.001**	<**0.001**
Social relationships	28.5%	22.8%	27.0%	0.529	**0.004**

a P-values were calculated using t-tests for continuous variables and chi-squared tests for categorical variables; bold font indicates p<0.05. At the time of affective disorder diagnosis (+ / - 6 months)

b Health of the Nation Outcome Scale 65+ (HoNOS65+) subscale scores 0-4 (0=least severe, 4 = most severe status) were categorised into scores of 0-1 representing that the patient was not experiencing problems in that domain, and scores of 2-4 representing experiencing problems. Values represent frequencies of patients scored as experiencing problems in that domain.

**Table 2:** **Factors associated with dementia development** in the three cohorts of patients with affective disorders using multivariate Cox proportional hazard model adjusted for age, age squared, gender, ethnicity, marital status, deprivation, baseline cognition and time*cognition interaction

	Bipolar disorder	Anxiety disorders	Depression
*Demographic variables*			
Age (per year increment)^a^	1.52 (0.70-3.32)	**1.84 (1.43-2.37)**	**1.57 (1.36-1.82)**
Female gender	0.51 (0.23-1.13)	0.92 (0.68-1.26)	1.02 (0.87-1.19)
Ethnicity			
White	1 (reference)	1 (reference)	1 (reference)
Black	1.46 (0.50-4.24)	1.30 (0.81-2.08)	**1.33 (1.08-1.64)**
Asian	2.03 (0.43-9.68)	1.05 (0.64-1.74)	1.15 (0.86-1.54)
Other	n/a	0.99 (0.46-2.12)	**1.70 (1.15-2.53)**
Married or cohabiting status^a^	0.77 (0.24-2.52)	1.17 (0.88-1.57)	1.09 (0.92-1.30)
Deprivation (per 10 unit increase in IMD score)^a^	1.20 (0.81-1.78)	1.03 (0.91-1.17)	1.02 (0.95-1.10)
Previous history of bipolar disorder/anxiety/depression	0.69 (0.16-2.91)	0.65 (0.15-2.76)	**0.62 (0.50-0.76)**
*HoNOS mental and physical health problems* ^a,b^			
Agitated behaviour	0.98 (0.45-2.14)	1.13 (0.78-1.62)	0.82 (0.66-1.03)
Non-accidental self-injury	0.40 (0.05-3.14)	0.87 (0.45-1.69)	**0.73 (0.56-0.94)**
Drug/alcohol problems	1.10 (0.24-5.09)	0.74 (0.36-1.53)	0.81 (0.56-1.17)
Hallucinations and delusions	**4.04 (1.75-9.33)**	**0.43 (0.21-0.90)**	0.82 (0.65-1.04)
Depressed mood	0.63 (0.20-1.95)	**0.65 (0.49-0.85)**	0.84 (0.71-0.99)
Cognitive problems	**5.62 (1.52-20.76)**	**4.64 (3.04-7.06)**	**3.94 (3.11-4.99)**
*HoNOS Physical illness or disability* (%)^a,c^			
No or minor	1 (reference)	1 (reference)	1 (reference)
Mild	0.95 (0.22-4.16)	0.73 (0.44-1.19)	0.91 (0.68-1.20)
Moderate - severe	1.21 (0.20-7.36)	**0.55 (0.33-0.91)**	0.76 (0.58-1.01)
*HoNOS functional problems* ^*a,b*^			
Activities of daily living	1.81 (0.78-4.19)	0.97 (0.72-1.30)	0.98 (0.83-1.15)
Social relationships	1.09 (0.47-2.52)	0.72 (0.49-1.05)	**0.80 (0.67-0.97)**

a at the time of affective disorder diagnosis (+ / - 6 months)

b Health of the Nation Outcome Scale 65+ (HoNOS65+) subscale scores 0-4 (0=least severe, 4 = most severe status) were categorised into scores of 0-1 representing that the patient was not experiencing problems in that domain, and scores of 2-4 representing experiencing problems. Values represent frequencies of patients scored as experiencing problems in that domain.

c Analysis incorporates a time*physical illness or disability interactions.

## Data Availability

All relevant aggregate data are found within the paper. The data use in this work have been obtained from the Clinical Record Interactive Search (CRIS), system that has been developed for use within the NIHR Mental Health Biomedical Research Centre (BRC) at the South London and Maudsley NHS Foundation Trust (SLaM). It provides authorised researchers with regulated access to anonymised information extracted from SLaM’s electronic clinical records system. Individual-level data are restricted in accordance to the strict patient led governance established at South London and The Maudsley NHS Foundation Trust, and by NHS Digital for the case of linked data. Data are available for researchers who meet the criteria for access to this restricted data: (1) SLaM employees or (2) those having an honorary contract or letter of access from the trust. For further details, and to obtain an honorary research contract or letter of access, contact the CRIS Administrator at cris.administrator@kcl.ac.uk.
